# Identification of Candidate Genes Associated With Hypoxia Tolerance in *Trachinotus blochii* Using Bulked Segregant Analysis and RNA-Seq

**DOI:** 10.3389/fgene.2021.811685

**Published:** 2021-12-14

**Authors:** Yifan Liu, Tian Jiang, Youming Chen, Yue Gu, Feibiao Song, Junlong Sun, Jian Luo

**Affiliations:** ^1^ State Key Laboratory of Marine Resource Utilization in South China Sea, Hainan Aquaculture Breeding Engineering Research Center, Hainan Academician Team Innovation Center, Hainan University, Haikou, China; ^2^ Hainan Blue Granary Technology Co., Ltd, Sanya, China

**Keywords:** Trachinotus blochii, hypoxia, tolerance, metabolism, BSR-seq

## Abstract

Golden Pompano (*Trachinotus blochii*) has rapidly developed into the one of the main valuable fish species in Chinese marine aquaculture. Due to its rapid growth, active metabolism, and high oxygen consumption, hypoxia will increase its mortality and cause serious economic losses. We constructed two experimental groups of fish with different degrees of tolerance to hypoxia, used BSR-Seq analysis based on genome and genetic linkage groups to locate SNPs and genes that were related to the differences in hypoxia tolerance. The results showed that hypoxia tolerance SNPs of golden pompano may be jointly determined by multiple linkage groups, especially linkage groups 18 and 22. There were 768 and 348 candidate genes located in the candidate regions of the brain and liver, respectively. These genes were mainly involved in anaerobic energy metabolism, stress response, immune response, waste discharge, and cell death. The *prostaglandin-endoperoxide synthase 2* (*PTGS2)* on LG8, which is involved in the metabolism of arachidonic acid, has a G/A nonsynonymous mutation at position 20641628, and the encoded amino acid was changed from hydrophobic aspartic acid to asparaginate. The specific pathway of the RIG-I-like receptor signaling pathway in the liver may mediate the metabolic system and the immune system, linking glucose metabolism with immune regulation. The death of the hypoxia-intolerant group may be due to the accumulation of lactic acid caused by the activation of anaerobic glycolysis during the early stage of hypoxia stress, and the activation of type I interferon was inhibited, which resulted in decreased immunity. Among the genes involved in the RIG-I-like receptor signaling pathway, the *CYLD Lysine 63 Deubiquitinase* (*CYLD)* located on LG16 had a G/T nonsynonymous mutation at position 13629651, and the encoded amino acid was changed from alanine acid to valine. The *interferon induced with helicase C domain 1* (*Ifih1)* located on LG18 has a G/C nonsynonymous mutation at position 16153700, and the encoded hydrophilic glycine was changed to hydrophobic alanine. Our findings suggest these SNPs may assist in the molecular breeding of hypoxia-tolerant golden pompano, and speculate that the balance of glucose and lipid metabolism plays a key role in *Trachinotus blochii* under acute hypoxia.

## Introduction

Oxygen is essential for most organisms as it plays a crucial role in aerobic metabolism (Richards, 2011) and dissolved oxygen (DO) in water is the basis of fish survival. However, oxygen content in the aquatic environment can fluctuate frequently, and environmental stress can impact fish greatly (Mahfouz et al., 2015; Zeng et al., 2016). Previous studies have shown that the DO levels in aquaculture waters should be maintained at over 4.0 mg/L (Song, 2009). Aquatic ecosystems are termed hypoxia when the concentration of DO drops below 2.0 mg/L (Diaz, 2001). Due to additional stresses from eutrophication, environmental pollution, and warming temperatures in recent years, hypoxia in aquatic systems is increasingly common, which may directly or indirectly change the structure and function of the aquatic ecosystem and threaten the development of fisheries (Diaz and Rosenberg, 2008; Breitburg et al., 2009). Therefore, the molecular mechanisms underlying fish response to hypoxia has become a subject of much interest in recent years (Mu et al., 2020).

In hypoxic or anoxic conditions, normal respiration and physiological metabolism of fish are disturbed, resulting in reduced food intake, a decrease in food conversion efficiency and slow growth, which have a negative impact on many important ecological and behavioral variables (Pichavant et al., 2000; Eby and Crowder, 2002; Aertebjerg et al., 2003). To maintain homeostasis and organism function in low-oxygen environments, fish have evolved many kinds of behaviors and physiological adaptation strategies. Hypoxia limits the exercise capacity of fish by limiting aerobic metabolism *in vivo* (Domenici et al., 2012), and slows the swimming rate to minimize energy consumption (Schurmann and Steffensen, 1994; Via et al., 1994; Herbert and Steffensen, 2005). Many fish respond to the effects of hypoxia by maintaining oxygen delivery with changes in blood indices, including the increase of red blood cells (RBC), hemoglobin (Hb) concentration, and serum iron content (SI), which play important roles in enhancing oxygen transport capacity (Soldatov, 1996; Affonso et al., 2002). In addition, fish can also improve the ability of their anaerobic metabolism to adapt to the hypoxia and prolong survival time (Chabot and Claireaux, 2008). Different species have different tolerance to hypoxia (Raaij et al., 1996; Ishibashi et al., 2002; Pichavant et al., 2002). Although there are interspecific differences, the physiological responses triggered by hypoxia in fish usually include primary hormonal responses, including catecholamines and/or cortisol followed by a series of related secondary responses including energy metabolism regulation, ion regulation, and cardiac ventilation regulation (Pickering and Pottinger, 1995; Mazeaud et al., 1977). In normoxia, the body mainly relies on aerobic respiration, with oxygen entering the mitochondria for oxidative phosphorylation and electron transport chain action. Nevertheless, under hypoxia stress, oxygen supply is insufficient and the oxidation of mitochondria cannot be completed, resulting in insufficient energy supply. To support normal requirements in an anoxic environment, vertebrate cells increase their anaerobic metabolism to provide energy, so the energy generation pathway will be converted from oxidative phosphorylation to anaerobic glycolysis (Barbour and Turner, 2014). The up-regulation of glycolytic enzymes, such as hexokinase (HK), phosphoglycerate kinase (PGK), and phosphoglycerate mutase (PGAM) is considered to be one of the key metabolic compensation mechanisms for hypoxia (Gao et al., 2015; Sun et al., 2017), and has been confirmed in the brain and heart of Danio rerio and the brain and liver of Pelteobagrus vachelli (Roesner et al., 2006; Zhang et al., 2017). Cells catabolize glucose to pyruvate *via* glycolytic enzymes (Marqueze et al., 2011). In oxygen-rich conditions, pyruvate is the primary carbon source and oxygen is the terminal electron acceptor in the Kreb’s cycle, which transfers electrons to the respiratory chain (Papandreou et al., 2006), with ATP as the end product. Lactate dehydrogenase (LDH) is a glycolytic enzyme that catalyzes the reversible conversion of lactate to pyruvate (Saavedra et al., 2016), which reflects the beginning of anaerobic respiration. A minority of fish also have the function of ethanol metabolism (Almeida-Val et al., 2011). *Carassius auratus*, a member of the Cypridae family, can increase the respiratory surface area and increase oxygen intake by changing the morphology of the gills, and at the same time discharge ethanol and carbon dioxide as the final product of glucose fermentation to avoid lactic acidosis (Nilsson and Renshaw, 2004; Nikinmaa and Rees, 2005).

Although fish can usually cope with short-term hypoxia, there are still differences between individuals (Sun et al., 2021). But it is difficult to accurately use these differences for hypoxia tolerance breeding. However, if reliable genetic markers are available, genetic improvement can be promoted. Identifying markers related to hypoxia tolerance is essential for applying marker-assisted selection (MAS) to improve hypoxia tolerance. Well-developed sequencing is revolutionizing molecular breeding by facilitating the development of a large number of single nucleotide polymorphism (SNP) markers (Dai et al., 2018). However, when the number of available SNP markers is not enough to implement MAS, RNA-seq analysis becomes an ideal alternative method, allowing markers to be found in expressed sequences (Lu et al., 2018). Michelmore first proposed Bulked Segregant Analysis (BSA) in 1991 (Michelmore et al., 1991), which is a practical gene marker mapping technology. Bulked Segregant RNA-Seq (BSR-Seq) is an efficient sequencing method that integrates BSA and RNA-Seq. It helps identify important chromosomal segments and finds the key genes that control different traits by detecting SNPs and calculating gene frequencies (Yao et al., 2017). This analysis method is based on the extreme phenotype of genotype. At present, this technology has been widely used to study complex traits such as growth and disease resistance in plants and animals (Wang et al., 2013; Du et al., 2017; Wang et al., 2017; Zhen et al., 2017; Dai et al., 2018).

Golden Pompano (*Trachinotus blochii*) has delicious meat without the intermuscular bone. It contains a variety of amino acids and fatty acids required by the human body, with high nutritional value and fast growth. The market demand for golden pompano is increasing at the moment, promoting interest in breeding (Ransangan et al., 2011). Adequate dissolved oxygen is critically important for its feeding, growth, disease resistance, and reproduction, but the golden pompano consumes large amounts of oxygen. In commercial production, increasing the stocking density can effectively increase the yield per unit water body. However, when the stocking density is too high, it can cause eutrophication which can increase the frequency and severity of hypoxia and eventually lead to the death of fish. In this study, we simulated hypoxia stress conditions and identified a hypoxia tolerant group (Tol group), and a hypoxia intolerant group (Intol group), of experimental fish. We used BSR-Seq analysis to identify and screen SNPs closely linked to differential genes of hypoxia tolerance. Based on the data of genome and genetic linkage groups, this work aims to provide new transcriptomics and SNPs, which will help develop molecular breeding of golden pompano tolerant to hypoxia and help to better understand the reasons for differences in hypoxia tolerance of *T. blochii*.

## Materials and Methods

### Experimental Fish

Hundreds of healthy golden pompano (50.0 ± 5.0 g) were obtained randomly from the Hainan Blue Ocean Aquaculture Co., Ltd. (Lingshui, Hainan, China) as the experimental subjects. After transporting them back to the laboratory, they were temporarily kept in 400 L tanks of circulating aerated seawater and in a stable water environment, i.e., temperature 27.0 ± 0.5°C; ammonia nitrogen and nitrite <0.02 mg/L; DO 7.0 ± 0.2 mg/L; pH 7.5 ± 0.2; salinity 20–30‰; and an equal photoperiod of 12 h of light and 12 h darkness. Commercial pellet feed (Tianma Company, Fujian, China) was fed daily in the morning and evening. The experimental fish were adapted to this experimental condition for 1 week. Feeding was stopped 24 h before the start of the formal experiment.

### Hypoxia Stress Experiment

We constructed three parallel tanks, each with a volume of 400 L, and randomly assigned 80 experimental fish to each tank. Initially all environmental conditions in each tank were kept the same as those of the temporary reception tanks. When the experiment started (t = 0 h), the DO was reduced to the level required for the experiment (i.e., 0.9 ± 0.1 mg/L) within 2 h by the introduction of nitrogen into the tanks. Under these conditions, the first 14 fish in each tank that exhibited raised heads and loss of balance within 1 h of hypoxic stress were taken as the hypoxic intolerant group (Intol). Similarly, the last 14 fish in each tank still exhibiting normal behavior after 24 h of hypoxic stress were taken as the hypoxic tolerant group (Tol). Water samples from each experimental tank were collected every hour. The DO level was monitored using the classic iodine quantity method (Beadle, 1958), and adjusted throughout the duration of the selection experiment in order to maintain the hypoxic conditions. The fish were fasted during this period.

### Sample Collection and RNA Isolation

After selection, as described above, fish were anesthetized with excess MS-222 (100 mg/L) which quickly killed the fish. In total, the Tol and Intol group each consisted of 42 experimental fish (14 per tank, three tanks). Brain and liver samples were taken from each fish, immediately frozen in liquid nitrogen and stored at −80°C until analysis. TRIzol reagent (Invitrogen, Carlsbad, CA, United States) was used, following the manufacturer’s instructions, to extract total RNA samples from the brain and liver of each fish and these were also stored at −80°C.

### RNA Library Construction and Sequencing

All RNA samples were standardized to 500 ng/μl, with associated quality parameters of: OD ratios of 260/280 and 260/230 greater than 1.8 and RNA Integrity Number (RIN) greater than 8.0. The 14 RNA samples obtained from each tank were mixed to construct RNA sequencing libraries, three for the Tol group and three for the Intol group, using a TruSeq™ RNA Sample Preparation Kit (Illumina Inc., San Diego, CA, United States). This was followed by KAPA quantification and dilution, after which each library was sequenced on an Illumina HiSeq 2500 (Illumina Inc.) with 125-bp paired-end reads. RNA-seq library preparation and sequencing was performed by BGI Technology Co., Ltd (Shenzhen, China).

### Discovery and Classification of DEGs

All read counts were normalized into FPKM. If the normalized expression of a gene was zero, it was modified to 0.01 for further analysis, and if the normalized expression of a gene for all samples was less than 1.0, it was removed from the differential expression analysis (Sun et al., 2019). Differential expression analysis was performed using the edgeR software package (version 3.8.2) (Robinson et al., 2010). The determination of differentially expressed genes (DEGs) must meet the following two criteria: | log2 (fold change) | >1 and *p* < 0.05. For this determination we used the OmicShare tool (www.omicshare.com/tools). DEGs were randomly selected for quantitative real-time PCR (qRT-PCR) to determine the accuracy of the sequencing data. Based on the sequencing, specific primers were designed using the Primer5 software (Supplementary Table S1). RNA samples were prepared and then reverse transcribed into cDNA. The reaction program for the 20 μl PCR system was 95°C for 20 s; 3 s at 95°C, 30 s at TM and 60 s at 72°C for 40 cycles, and all samples were performed in triplicate. β-actin expression was used to determine the relative expression of genes, and the 2^−ΔΔCT^ method was used to analyze the gene expression level (Livak and Schmittgen, 2001).

### Clean Reads Filtering

Four raw reads were obtained by merging three parallel raw data streams from the Intol and Tol brain and liver samples. These raw reads contained a lot of redundant and unqualified sequences that needed to be filtered out. The filtering software SOAPnuke was used for statistics, and then the Trimmomatic module was used for filtering. After removing adaptor sequences, ambiguous N nucleotides (i.e., those with an N ratio >5%), and low-quality sequences (i.e., reads with <50% bases of quality value), the remaining clean reads were assembled using the Trinity software package (Grabherr et al., 2011).

### Genome Alignment, Variants Identification and Filtering

To identify SNPs and InDels, filtered reads were aligned to the reference genome using the Burrows-Wheeler Aligner (BWA, v 0.7.16a-r1181) with the parameter “mem -M,” -M is an option used to mark shorter split alignment hits as secondary alignments (Li and Richard, 2009). Then Picard software was used to sort alignment results and mark repeated sequences. The reference genome version is available but has not yet been published.

Variant calling was carried out using GATK UnifiedGenotyper (v3.5). SNPs and InDels were filtered using the GATK VariantFiltration function with proper standards (-Window 4,-filter “QD < 4.0 || FS > 60.0 || MQ < 40.0,”-G_filter “GQ < 20”). Several additional conditions were used for further marker filtering, i.e.: *1*) markers with any missing genotypes were excluded; *2*) markers with reading depth <10X or >500X in per bulk were excluded to eliminate those with low confidence due to low coverage, or those that may be in repetitive regions and thus have inflated read depths; *3*) markers with SNP-index values in both bulks <0.3 or >0.7 were excluded.

### Calculation of Target Candidate Regions

Here we used commonly applied methods for BSR-Seq analysis, including the SNP-index method, the Euclidean Distance (ED) method and the G statistic method.

1) Calculation of SNP-index and ∆(SNP-Index)

The SNP-index and the ∆(SNP-Index) (Takagi et al., 2013) of each SNP/InDel was calculated as follows:

SNP-index = *AD*_*r*_/(*AD*_*d*_+*AD*_*r*_)

*AD*, Average depth of mixed pool samples; *AD*_*r*_, Average depth of recessive/mutation bulk; *AD*_*d*_, Average depth of dominance/wild bulk.

∆(SNP-Index) = SNP-index(recessive/mutation bulk) − SNP-index(dominance/wild bulk).

2) Calculation of G statistic

The formula used to calculate the G statistic of each SNP was:

$$G=2\sum_{i}Oi\times \ln\left(O_{i}/E_{i}\right)$$

Where *O* is the observed *AD* (*AD*_*r*_, *AD*_*d*_), E is the expected AD under the null hypothesis and is calculated as in the original G-statistic method (Magwene et al., 2011), and ln denotes the natural logarithm.

3) Calculation of Euclidean Distance (ED)

The modified ED-value calculation formula used was:

ED=(Altr−Altd)2+(Refr−Refd)2

Where *Alt*_*r*_, *Alt*_*d*_, *Ref*_*r*_ and *Ref*_*d*_ represent non-reference allele depth in recessive/mutation bulk, non-reference allele depth in dominance/wild bulk, reference allele depth in recessive/mutation bulk, reference allele depth in dominance/wild bulk, respectively. To eliminate the background noise, all ED values were powered, and the ED5 was used as the final ED value (Hill et al., 2013).

### Ontology and Enrichment Analysis

GO and KEGG comparisons were performed using the ultrageometric test to identify which DEGs were significantly enriched in GO terms (*p* < 0.05) and KEGG pathways (*p* < 0.05) compared with the whole transcriptome background. The KEGG database was used to integrate the focal pathways (Kanehisa et al., 2017), and integrate these pathways according to whether the gene expression pattern is consistent with the upstream and downstream gene expression trends of the pathway (Sun et al., 2020).

## Results

### Sequence Assembly and Alignment Analysis

A total of 1,082.51 (M) raw reads (150 bp) were obtained from 12 mixed samples of different groups on the Illumina HiSeq platform. After integrating raw reads and filtering the low-quality sequences, the clean read count was 978.64 (M). The clean reads of four samples were aligned with the reference genome, and high-quality alignment results were obtained (Table 1). The total mapped rate of all samples was greater than 88.39%, and the unique mapped rate was higher than 86%. This indicated that the mixed pool was relatively similar to the reference sequence, and also indicated the integrity of the reference sequence. In addition, the multiple mapped rate was less than 6%, which showed that the number of highly similar genes in the golden pompano genome was limited. Therefore, in order to ensure the accuracy of the alignment, clean reads with unique mapped positions were selected for subsequent variant calling.

**TABLE 1:** Statistical analysis of alignment results with reference genome.

Group	Sample	Total read	Total mapped	Unique mapped	Multi-mapped
Intol	Brain	247623544	221011216 (89.25%)	215783989 (87.14%)	5227227 (2.11%)
	Liver	241424998	224096809 (92.82%)	211216836 (87.49%)	12879973 (5.33%)
Tol	Brain	247282594	218582347 (88.39%)	213513072 (86.34%)	5069275 (2.05%)
	Liver	242207708	226191931 (93.39%)	214205070 (88.44%)	11986861 (4.95%)

### Analysis of DEGs During Hypoxia

**FIGURE 1:**
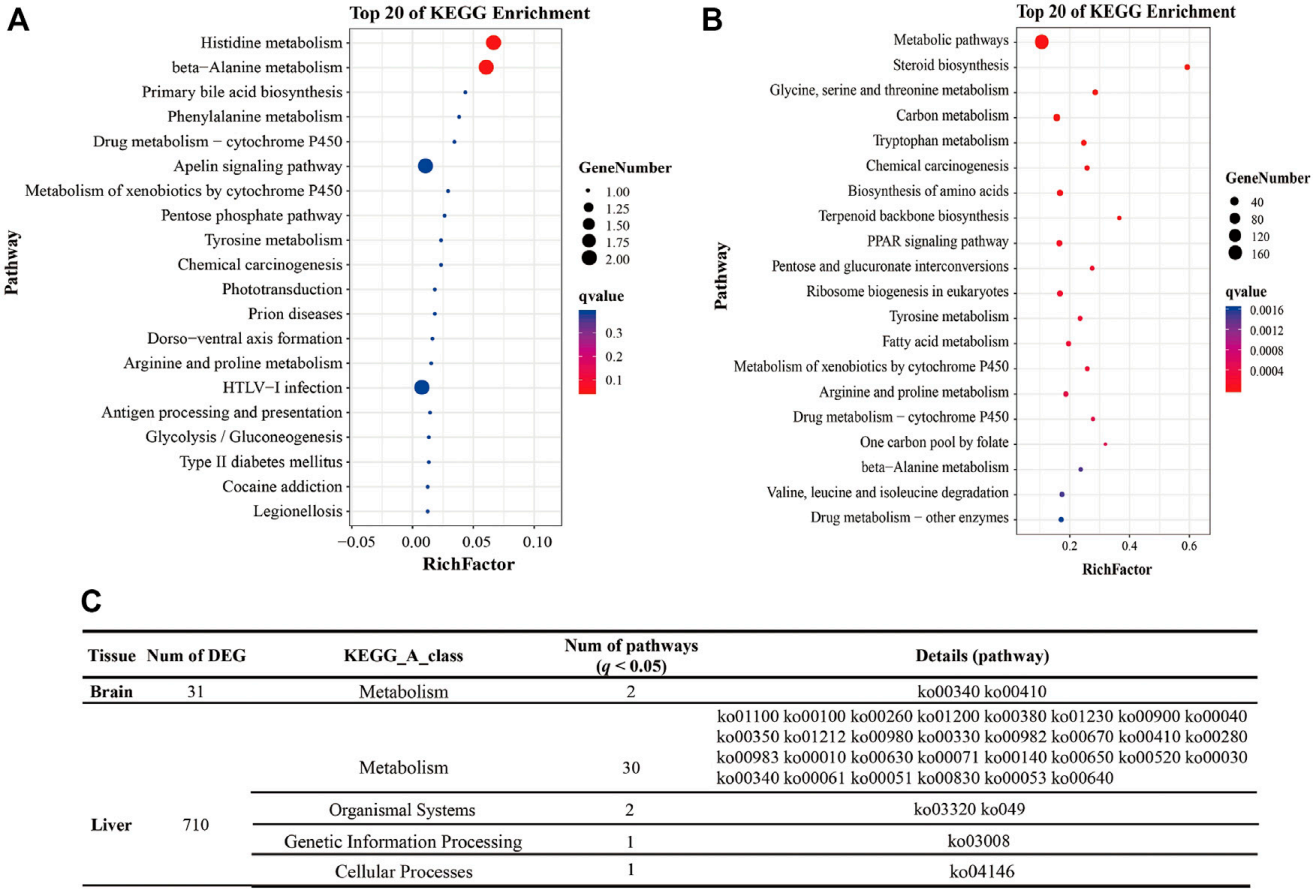
Pathway enrichment analysis of DEGs. Note: **(A)** The top 20 pathways enriched by DEGs of the brain in the different groups (Intol-vs-Tol). **(B)** The top 20 pathways enriched by DEGs of the liver in the different groups (Intol-vs-Tol). **(C)** The table shows the statistical results of pathways with *q* < 0.05.

A previous study of ours described the mRNA profile from the brain and liver of golden pompano (Sun et al., 2021). The results of RT-qPCR showed a significant positive correlation with the RNA-Seq results (*p* < 0.05) (Supplementary Figure S1), indicating that our sequencing results were accurate. First, a total of 31 DEGs and 710 DEGs were significantly differentially expressed in the brain and liver in different groups. For more information about DGEs expression levels, *see* Supplementary Figure S1. Only two pathways (Histidine metabolism, beta-Alanine metabolism) were significantly enriched in the brain (*q* < 0.05). However, 77 DEGs were classified into carbohydrate and lipid metabolism processes, including Pentose and glucuronate interconversions, Glycolysis/Gluconeogenesis, Glyoxylate and dicarboxylate metabolism, Butanoate metabolism, Amino sugar and nucleotide sugar metabolism, Pentose phosphate pathway, Fructose and mannose metabolism, Ascorbate and aldarate metabolism, Propanoate metabolism, Steroid biosynthesis, Steroid hormone biosynthesis, Fatty acid degradation and biosynthesis (Figure 1 and Supplementary Figure S2).

### Determination of the Candidate Area

**FIGURE 2:**
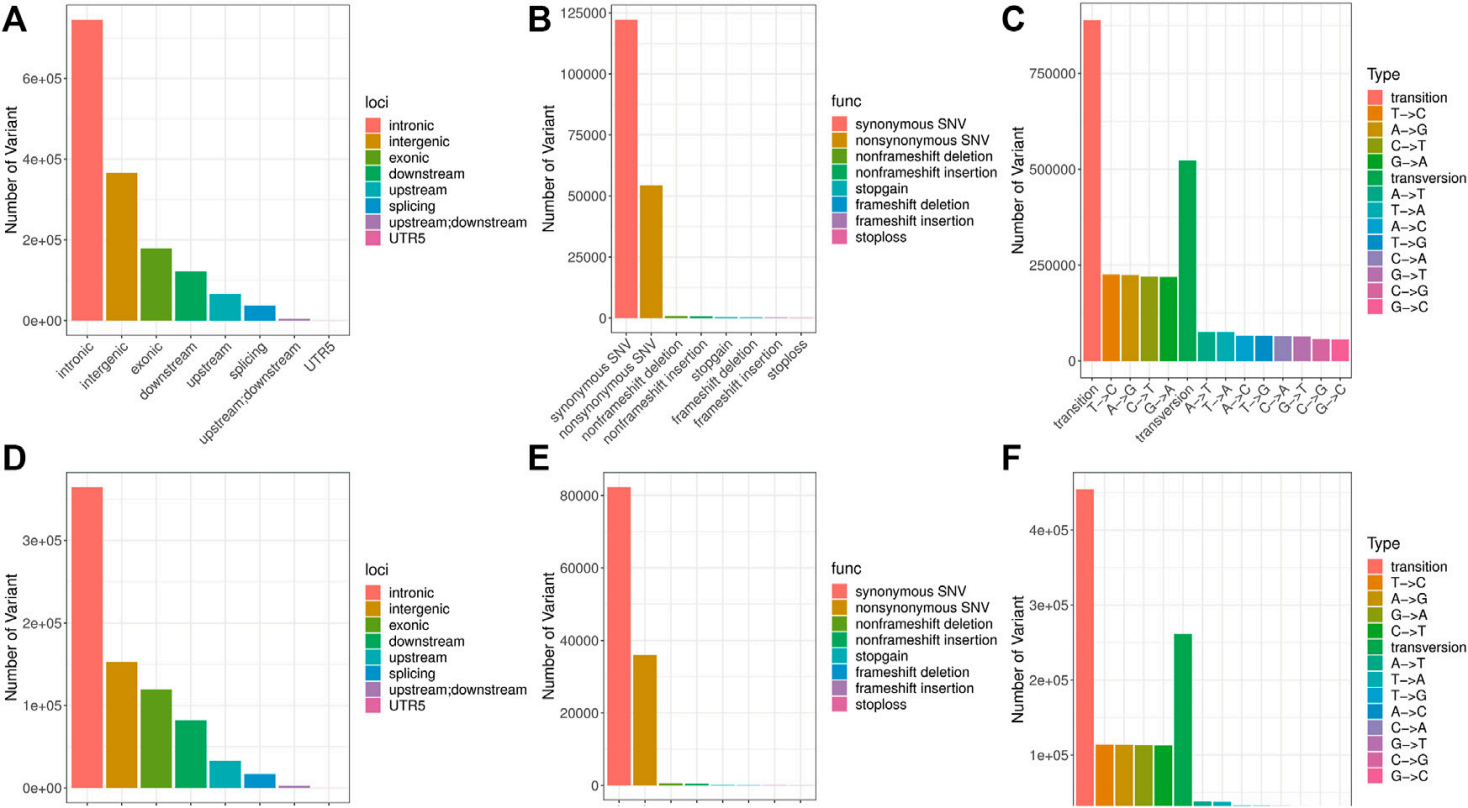
Location information, function note and type distribution of SNPs. Note: **(A**–**C)** Location information, function note and type distribution of SNPs in the brain. **(D**–**F)** Location information, function note and type distribution of SNPs in the liver.

A total of 1,411,727 SNPs and 106,475 InDels were detected in the brain, and a total of 770,770 variants were detected in the liver, including 715,889 SNPs and 54,881 InDels (Table 2). These SNP/InDels were distributed on the 24 linkage groups of golden pompano. In the brain, there were 122,176 SNP/InDels with synonymous mutations in the exon region and 54,333 SNP/InDels with non-synonymous mutations (Figures 2A–C). In the liver, there were 32,656 SNP/InDels located in the upstream region, 152,863 SNP/InDels located in the exon region, 82,267 SNP/InDels with synonymous mutations, and 35,969 SNP/InDels with non-synonymous mutations (Figures 2D–F). After filtering through a series of thresholds, the test results showed that 555,478 SNPs and 65,157 InDels were obtained in the brain, and 265,920 SNPs and 30,684 InDels were obtained in the liver (Supplementary Figure S3 and Supplementary Figure S4).

**TABLE 2:** Statistical table of variation number before and after filtering.

Sample	Variantion	Before filter	After filter
Brain	Total	1518202	620635
	SNP	1411727	555478
	Indel	106475	65157
Liver	Total	770770	296604
	SNP	715889	265920
	Indel	54881	30684

#### BSR-Seq Analysis Based on SNP-Index Method

**FIGURE 3:**
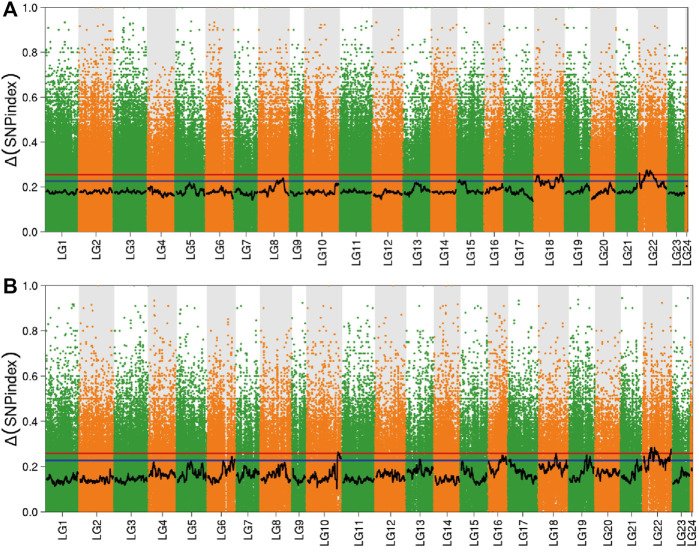
Distribution of Δ (SNP-index) in the brain and liver. Note: **(A)** Brain; **(B)** Liver; The red line in the figure is the 99% confidence level line, and the blue line is the 95% confidence level line.

In order to determine the reasonable fluctuation range of SNP-index and Δ (SNP-index), we performed 10,000 simulations on the ratio of dominant and recessive alleles based on population type, mixed pool size, and sequencing depth. After simulation, 95% (SNP-index) and 99% (Δ (SNP-index)) confidence intervals in the simulated values were taken as reasonable fluctuation ranges. After fitting, values exceeding the range were regarded as significant values, and the intervals corresponding to the significant values were regarded as candidate intervals. Combining this with the distribution of Δ (SNP-index) on the linkage group allowed the Manhattan scatter plot to be drawn (Figure 3). In the brain, there were eight significant intervals obtained by screening, using a 99% confidence interval, which were distributed in linkage groups 6, 7, 12, 17, 18, 20, and 22. However, in the liver, a 99% confidence interval obtained 10 significant intervals, which were distributed in linkage groups 1, 2, 3, 6, 9, 11, 14, 15, 18, and 22.

#### BSR-Seq Analysis Based on G Statistic Method

**FIGURE 4:**
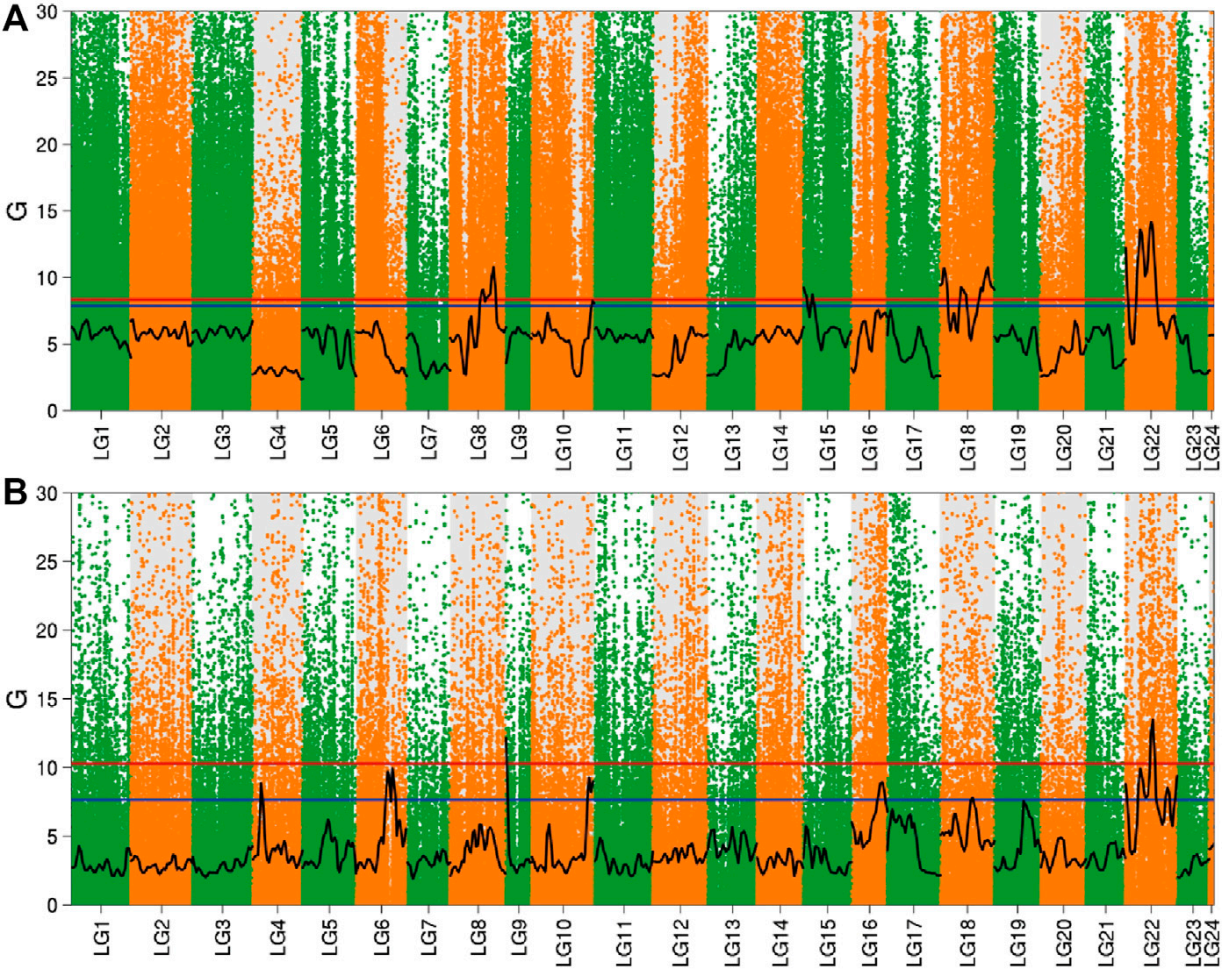
Distribution of G value in the brain and liver. Note: **(A)** Brain; **(B)** Liver; The red line in the figure is the 99% confidence level line, and the blue line is the 95% confidence level line.

After fitting with the cube method, the *p* values of each site were calculated by combining the Hampel rule and the Log-normal distribution. The *p* value was corrected using FDR to obtain the q value, and 0.01 and 0.05 were used as the threshold values, respectively. Combined with the distribution of the G value on the linkage group, the Manhattan scatter plot was drawn (Figure 4). In the brain, there were four significant intervals obtained by screening according to a 99% confidence interval, which were distributed in linkage groups 8, 15, 18, and 22. However, in the liver, selected using 99% as the confidence interval, two significant intervals were obtained which were distributed in linkage groups 9 and 22.

#### BSR-Seq Analysis Based on Euclidean Distance (ED) Method

**FIGURE 5:**
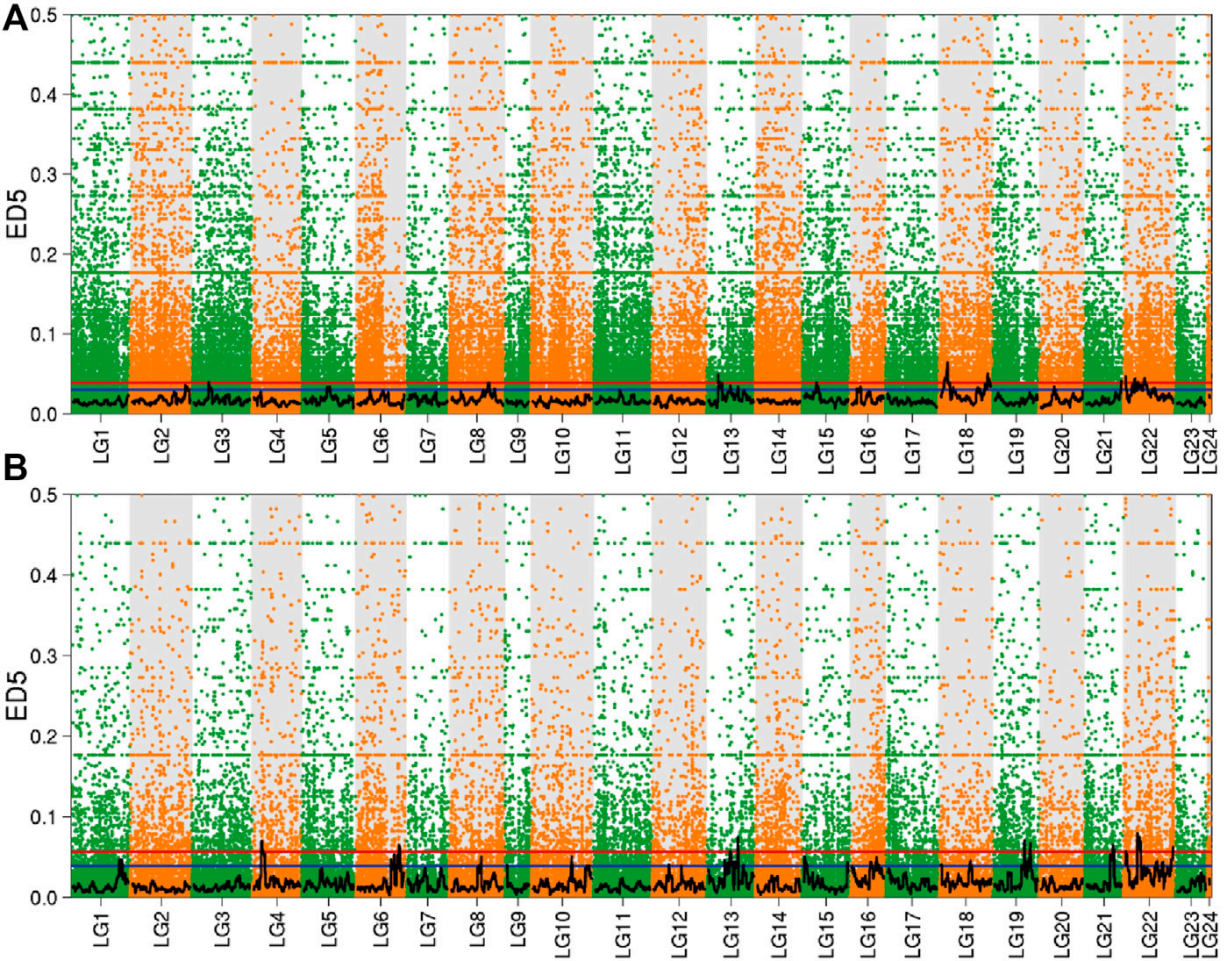
Distribution of ED value in the brain and liver. Note: **(A)** Brain; **(B)** Liver; The red line in the figure is the 99% quantile line, and the blue line is the 95% quantile line.

The significant interval obtained by screening the 95 and 99% quantiles, and combined with the distribution of ED value on the linkage group to draw the Manhattan scatter plot (Figure 5). In the brain, there were 10 significant intervals obtained by screening according to 99% confidence interval, which were distributed in linkage groups 3, 8, 13, 15, 18, 21, and 22. However, in the liver, selected 99% as the confidence interval to obtain six significant intervals, which were distributed in linkage groups 4, 6, 13, 19, 21, and 22.

### Integration Analysis of Candidate Regions and Screening of Candidate Genes

Here, we used the intersection of three positioning methods. The positioning intervals of each method was small under 99% conditions, and the intersection of the three positioning intervals could not be obtained. However, under 95% conditions, eight significant intervals were found in the brain, which were located on linkage groups 8, 18, and 22. Among them, there were two significant intervals on linkage group 8, and three significant intervals on linkage group 18 and 22 respectively (Table 3). Taking the genes in these intervals as candidate genes, there were 768 candidate genes in the brain (Supplementary Figure S5).

**TABLE 3:** Statistical results of brain and liver candidate regions.

Tissue	Linkage group	Starting position	Ending position	Length(Mb)
Brain	LG8	19000001	21890000	2.89
	LG8	22380001	23900822	1.52
	LG18	1170001	3916209	2.75
	LG18	19510344	22340000	2.83
	LG18	22940001	28154750	5.21
	LG22	92365	1510067	1.42
	LG22	5545435	13640000	8.09
	LG22	14120001	16534294	2.41
Liver	LG10	28905222	31200000	2.29
	LG16	13597064	16040000	2.44
	LG18	15904147	16992561	1.09
	LG22	5123	938598	0.93
	LG22	6489553	10120000	3.63
	LG22	15630001	15964972	0.33
	LG22	20987572	21760000	0.77
	LG22	25719411	26872637	1.15

There were eight significant intervals in the liver, which were located on linkage groups 10, 16, 18, and 22. Among them, there was one significant interval on linkage groups 10, 16, and 18. While the number of significant intervals on the 22 linkage group was the largest, with a total of 5 (Table 3), The total number of candidate genes in the interval was 348 (Supplementary Figure S5). It is speculated that the hypoxia tolerance of golden pompano may be jointly determined by multiple linkage groups. After comparing the candidate genes in the brain and liver, it was found that a total of 140 genes were located simultaneously in the candidate interval of both brain and liver (Supplementary Table S2).

### Functional and Pathway Enrichment of Candidate Genes

**FIGURE 6:**
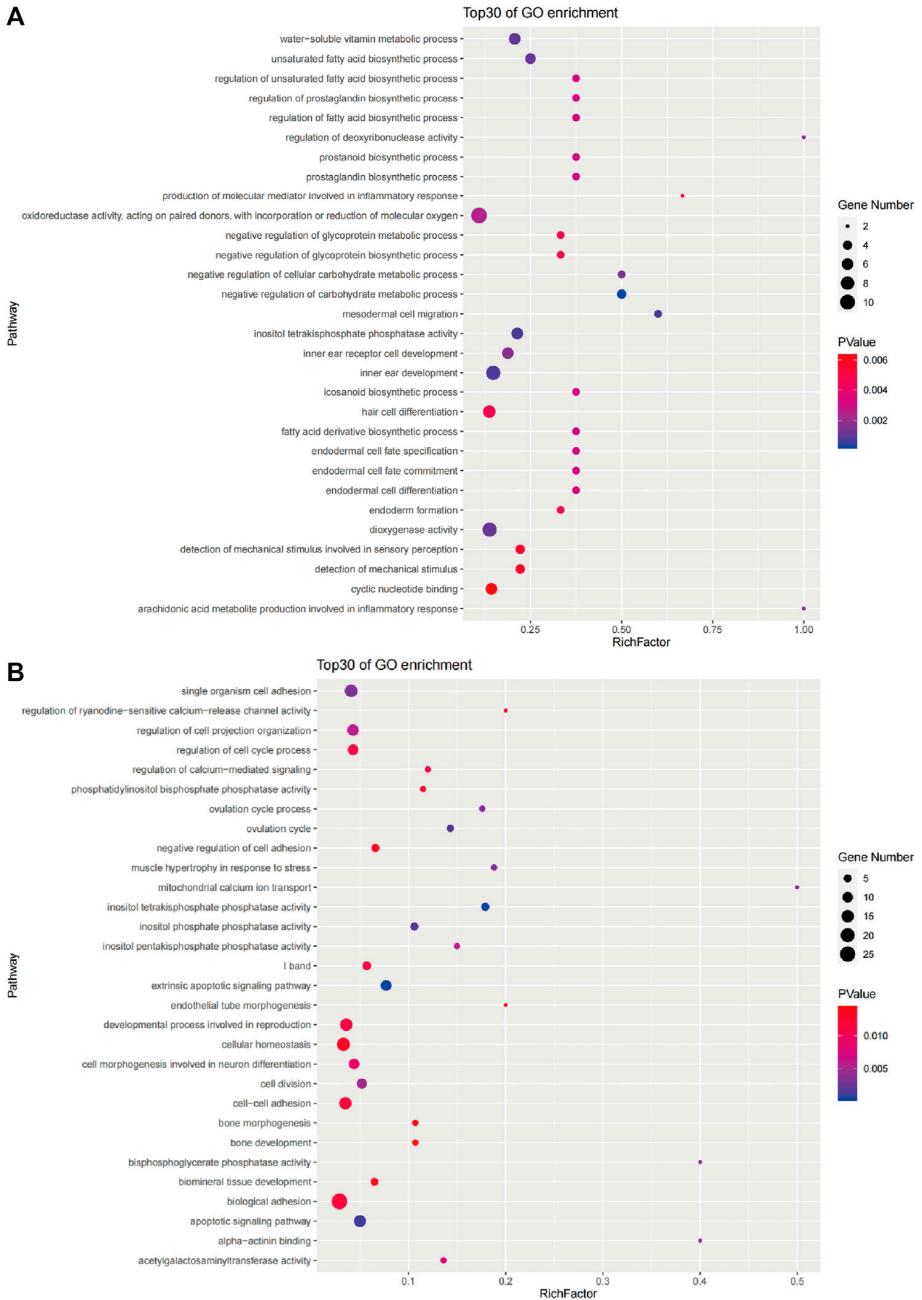
The top 30 GO terms enriched by candidate genes of the brain **(A)** and liver **(B)**.

We screened the candidate genes in the brain for GO and KEGG classification analysis (Supplementary Figure S6 and Supplementary Figure S7). A total of 768 candidate genes in the brain were assigned to 166 level2_GO sub-categories (*p* < 0.05) (Supplementary Figure S2), and there were 30 main GO terms (Figure 6A). Among these, four negative regulation of carbohydrate metabolic process were the highest enrichment terms, followed by three mesodermal cell migration, and six inositol tetraphosphate phosphatase activity. In addition, the number of genes classified as cytoplasm was the largest in the significantly enriched terms. From the GO enrichment analysis, it can be seen that the process of hypoxia tolerance in golden pompano was mainly closely related to cell structure, migration, and energy metabolism.

**FIGURE 7:**
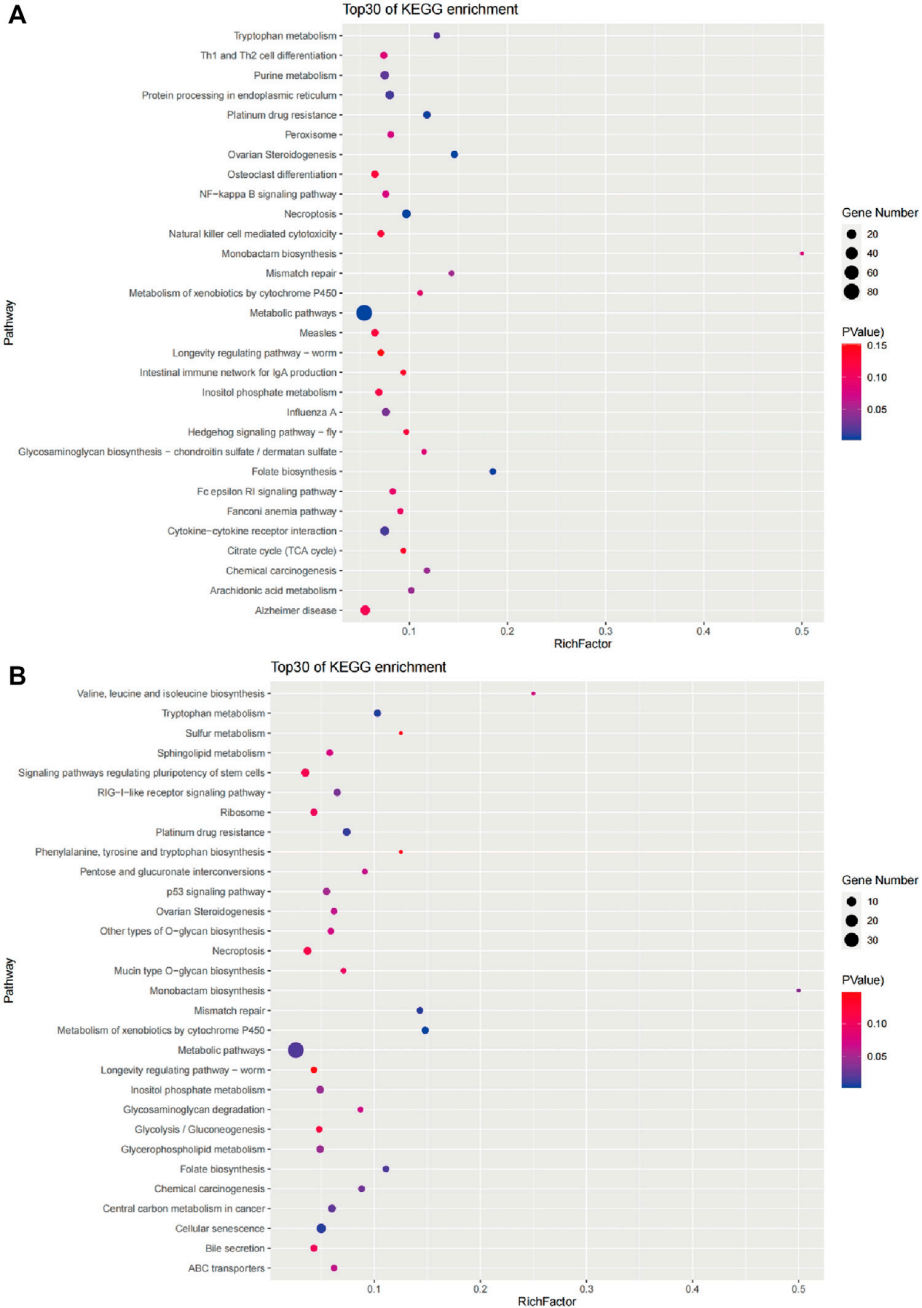
The top 30 pathways enriched by candidate genes of the brain **(A)** and liver **(B)**.

A KEGG pathway analysis was performed for 768 candidate genes in the brain to identify the biochemical pathways operating. The results revealed 300 enriched pathways, and 12 pathways were significantly enriched (*p* < 0.05). The 30 main pathways are shown in Figure 7A and include cellular processes, genetic information processing, environmental information processing, metabolism, organismal systems, and human diseases (Table 4). Among them, the number of genes involved in the metabolism pathway was the largest, including Metabolic pathways (ko01100), Folate biosynthesis (ko00790), Tryptophan metabolism (ko00380), Purine metabolism (ko00230), and Arachidonic acid metabolism (ko00590).

**TABLE 4:** Statistical table of significant enrichment pathway of candidate genes.

	Num of genes	KEGG_A_Class	Pathway(*p* < 0.05)
Brain	768	Metabolism	ko01100 ko00790 ko00380 ko00230 ko00590
		Genetic Information Processing	ko04141
		Celluar Process	ko04217
		Environmental Information Processing	ko04060
		Organismal Systems	ko04913
		Human Diseases	ko01524 ko05164 ko05204
Liver	348	Metabolism	ko00980 ko00380 ko00790 ko01100 ko00261 ko00562 ko00564
		Genetic Information Processing	ko03430
		Celluar Process	ko04218 ko04115
		Organismal Systems	ko04622
		Human Diseases	ko01524 ko05230 ko05204

We screened the candidate genes in the liver for GO and KEGG classification analysis (Supplementary Figure S6 and Supplementary Figure S7). A total of 348 candidate genes in the liver were assigned to 149 level2_GO sub-categories (*p* < 0.05) (Supplementary Figure S3); there were 30 main GO terms shown in Figure 6B. Among them, 10 extrinsic apoptotic signaling pathways were the highest enrichment terms, followed by five inositol tetrakisphosphate phosphatase activity, and 13 apoptotic signaling pathways. In addition, the number of genes classified as the membrane was the largest in the significantly enriched terms. From the GO enrichment analysis, it can be seen that the process of hypoxia tolerance in golden pompano was mainly closely related to cell apoptosis.

A KEGG pathway analysis was performed for 348 candidate genes in the liver to identify the biochemical pathways operating. The results revealed 233 enriched pathways, and 14 pathways were significantly enriched (*p* < 0.05). The 30 main pathways are shown in Figure 7B and include cellular processes, genetic information processing, metabolism, organismal systems, and human diseases (Table 4). Among them, the number of genes involved in the metabolism pathway was the largest, including Metabolism of xenobiotics by cytochrome P450(ko00980), Tryptophan metabolism (ko00380), Folate biosynthesis (ko00790), Metabolic pathways (ko01100), and Monobactam biosynthesis (ko00261), Inositol phosphate metabolism (ko00562), Glycerophospholipid metabolism (ko00564).

### Significant SNPs Associated With Hypoxia Stress

**FIGURE 8:**
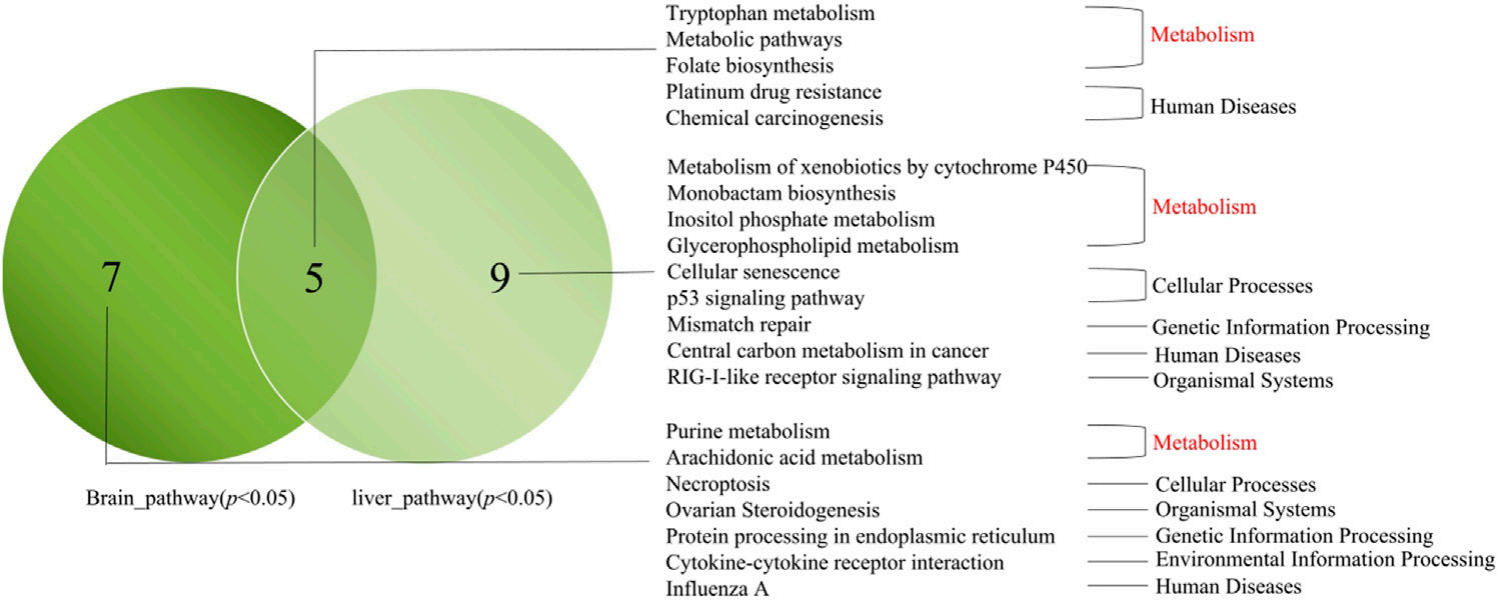
Venn map of pathways between the brain candidate genes in the brain and liver. Note: Delineated within the dark green circle was the pathways which enriched for candidate genes in the brain (*p* < 0.05). Delineated within the light green circle was the pathways which enriched for candidate genes in the liver (*p* < 0.05). The middle cross section showed the common pathways enriched. The main pathways were listed in this figure.

Based on the candidate genes in the brain and liver of the different groups, we analyzed the significantly enriched pathways in the brain and liver (*p* < 0.05) (Figure 8). In short, we found five similar pathways in the brain and liver, including three metabolic pathways (Tryptophan metabolism, Metabolic pathway, and Folate biosynthesis). Besides, seven pathways were significantly enriched only in the brain (Purine metabolism, Arachidonic acid metabolism, Necroptosis, Ovarian Steroidogenesis, Protein processing in endoplasmic reticulum, Cytokine-cytokine receptor interaction, and Influenza A). Nine pathways were only significantly enriched in the liver (Metabolism of xenobiotics by cytochrome P450, Monobactam biosynthesis, Inositol phosphate metabolism, Glycerophospholipid metabolism, Cellular senescence, p53 signaling pathway, Mismatch repair, Central carbon metabolism in cancer, and RIG-I-like receptor signaling pathway).

We further speculate that the arachidonic acid metabolism pathway and RIG-I-like receptor signaling pathway may play an important role in acute hypoxia stress of the golden pompano. Here, there are five important genes involved in the arachidonic acid metabolism pathway among the candidate genes, which are Trachinotus_GLEAN_10010123 (*PTGS2*), Trachinotus _GLEAN_10010124 (*pla2g4a*), Trachinotus_GLEAN_10015446 (*ALOX5*), Trachinotus_GLEAN _10015447 (*ALOX5*) and Trachinotus_GLEAN_10017022 (*PLA2G12B*). Among these important genes, the *PTGS2* gene located on LG8 has a G/A nonsynonymous mutation at position 20641628, and the encoded amino acid was changed from hydrophobic aspartic acid to asparaginate (Supplementary Figure S8). There are four important genes involved in the RIG-I-like receptor signaling pathway among the candidate genes, which are Trachinotus_GLEAN_10002391 (*CYLD*), Trachinotus_GLEAN_10017046 (*trim72*), Trachinotus_GLEAN_10018773 (*Ifih1*), and Trachinotus _GLEAN_10018778 (*TANK*). Among these important genes, the *CYLD* gene located on LG16 has a G/T nonsynonymous mutation at position 13629651, and the encoded amino acid was changed from alanine acid to valine. The *Ifih1* gene located on LG18 has a G/C nonsynonymous mutation at position 16153700, the encoded amino acid was changed from hydrophilic glycine to hydrophobic alanine (Supplementary Figure S8). By predicting the gene domains of *PTGS2*, *CYLD,* and *Ifih1*, it was found that the region of 20641628 G/A variant site of *PTGS2* is specifically matched with the protein domain of prostaglandin-endoperoxide synthase, and the region of 13629651 C/T variant site of *CYLD* is specifically matched with the CAP-Gly domain.

### Differentially Expressed Genes Between Bulks

**FIGURE 9:**
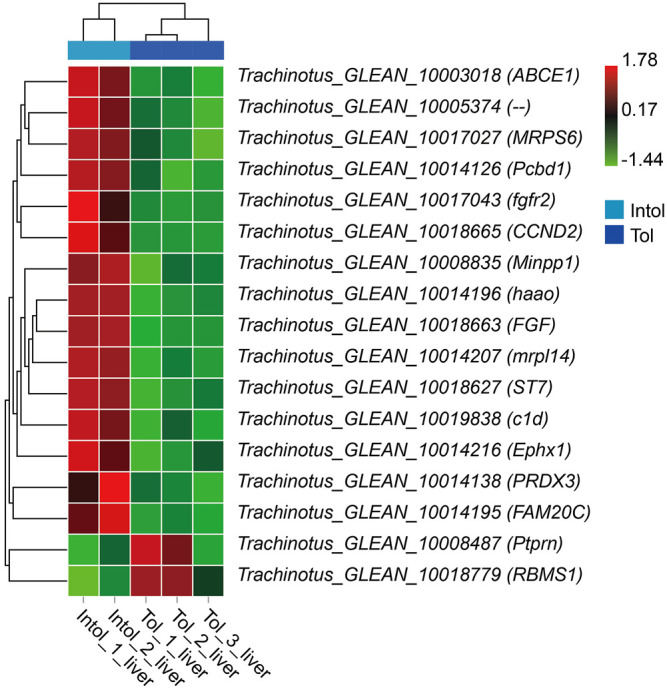
Heat map of 17 differentially expressed candidate gene expression patterns.

When integrated with the results of BSA and transcriptome analysis, there is no difference in the expression of 768 candidate genes in the brain. However, 17 of the 348 candidate genes in the liver obtained by BSR-Seq analysis were differentially expressed genes (Figure 9). The annotated results of these genes showed that the *Minpp1*, *Pcbd1*, *haao*, *Ephx1*, and *RBMS1* were classified into the metabolism pathways.

## Discussion

The traditional method of mining SNP related to target traits, if there is no reference genome, will be time-consuming and inefficient (Lu et al., 2016). Instead, coupling of RNA-seq with BSA can allow correlation of global expression patterns and SNPs with a target trait. For example, the application of this technology was used to study catfish resistance to intestinal sepsis and identified 1,255 DEGs and 56,419 SNPs which have a significant allelic imbalance between resistant and susceptible groups (Wang et al., 2013).

To our knowledge, ours is the first study of SNPs related to hypoxia tolerance in the golden pompano (*Trachinotus blochii*) using BSA along with pooled RNA-seq. First, clean reads were compared with the reference genome, and the number of trusted reads that were compared to a unique position accounted for more than 86%. After mutation detection and screening, a total of 555,478 SNPs and 65,157 InDels were detected in the brain, and a total of 265,920 SNPs and 30,684 InDels were detected in the liver. Integrating the results of the SNP-index analysis method, Gstatistic method, and ED analysis method, it was found that candidate regions in the brain were located in linkage groups 8, 18, and 22, and candidate regions in the liver were located in linkage groups 10, 16, 18, and 22. It is speculated that hypoxia tolerance of golden pompano may be determined by multiple linkage groups, especially linkage groups 18 and 22. In our experiments, BSR-Seq analysis identified 768 candidate genes in the brain and 348 candidate genes in the liver. We annotated these candidate genes to gain an insight into the biological processes and pathways of the key genes involved in the hypoxia adaptation of golden pompano*.* These pathways were involved in anaerobic energy metabolism, stress response, immune response, waste discharge, and cell death, enhancing the hypoxia tolerance and survival ability of fish. These results are consistent with previous research on other fish species, such as ruffe (*Gymnocephalus cernua*), flounder (*Platichthys flesus*) (Tiedke et al., 2014) and darkbarbel catfish (*Pelteobagrus vachelli*) (Zheng et al., 2021).

Our previous research results show that the brain and liver exhibit a synergistic response to promote adaptation to hypoxia, and that disorder of liver glucose and lipid metabolism under hypoxia stress may lead to the death of golden pompano (Sun et al., 2021). Similar results were also found in this study using BSR-seq technology. Here, we found 17 differently expressed candidate genes, and completely opposite expression patterns were identified. The annotated results of these genes showed that the *Minpp1*, *Pcbd1*, *haao*, *Ephx1*, and *RBMS1* were classified as the metabolism pathways (Duignan et al., 1988; Sun et al., 2019). It is speculated that the differences in the expression and sequence of these genes may affect the ability of golden pompano to respond to acute hypoxia stress.

Previous experiments have indicated indirectly that arachidonic acid has a regulatory effect on lipid accumulation in fish. For example, studies have shown that dietary arachidonic acid can effectively reduce lipid accumulation in juvenile grass carp (*Ctenopharyngodon idella*) (Tian et al., 2014) by inducing lipolysis and inhibiting adipogenesis (Tian J.-j. et al., 2017). Additionally it has been shown that COX-mediated metabolites play important roles in the inhibition of lipid accumulation in the hepatopancreas of grass carp fed with ARA (Tian J.-J. et al., 2017). Other researchers have found that arachidonic acid can reduce the lipid content in the liver of juvenile *Synechogobius hasta* (Luo et al., 2012) and gilthead sea bream (Sparus aurata L.) (Fountoulaki et al., 2003), as well as whole-body lipid content of juvenile Japanese seabass (Laeolabrax japonicus) (Xu et al., 2010). COX encoded by the PTGS2 gene on arachidonic acid metabolism catalyzes the conversion of arachidonic acid into prostaglandins (PGs) (Radi, 2009), among which PGE_1 can inhibit cholesterol biosynthesis. This may explain that the region of the 20641628 G/A variant site of *PTGS2* is specifically matched with the protein domain of prostaglandin-endoperoxide synthase. Thus, we conclude that arachidonic acid may promote lipid metabolism and energy supply by reducing lipid accumulation, thereby improving the adaptability of golden pompano to hypoxia.

When fish cannot produce enough energy to meet their needs through aerobic metabolism under hypoxic conditions, they will be forced to use their anaerobic metabolism (Polymeropoulos et al., 2017). The switch to anaerobic respiration involves the utilization of glucose through the glycolysis pathway (Mandic et al., 2013). Glycolysis is a ubiquitous glucose degradation pathway in all biological organisms (Corkins et al., 2017). Hypoxia is perhaps the most physiologic inducer of p53 (Graeber et al., 1994), and hypoxia-mediated apoptosis *in vivo* requires p53 (Graeber et al., 1996). In addition, p53 has a certain effect on the regulation of cellular energy metabolism signaling pathways in hypoxia (Feng and Levine, 2010). In our results, there are SNPs in some genes of the P53 signaling pathway in the liver, hence it may be that the p53 signaling pathway plays an important role in the hypoxia of golden pompano.

It has been reported that in order to adapt to short-term hypoxia stress, the expression of genes related to oxidative phosphorylation, glycolysis and glycogenolysis pathways in fish will be significantly increased (McBryan et al., 2016; Sun et al., 2020). In the early stage of stress, carbohydrate decomposition and oxidative phosphorylation are used to maintain the balance between supply and demand of ATP *in vivo* (Everett et al., 2011). The expression of genes related to the glycolysis/gluconeogenesis pathway in the liver of the adult Nile tilapia (*Oreochromis niloticus*) and the oxidative phosphorylation pathway in the liver of the Gulf killifish (*Fundulus grandis*) were significantly increased under short-term hypoxia stress (Everett et al., 2011; Xia et al., 2017; Li et al., 2018).

It is well known that there is a close connection between the energy-supplying metabolic system and the immune system. Previous studies have found that when the RLRs (RIG-I like receptors) in cells are stimulated, RLRs can activate RIG-I-like receptor signaling pathways associated with a viral infection (Zhang et al., 2019). During the activation, the process of glycolysis is inhibited, and the key to this inhibitory effect is the production of type I interferon, which initiates the immune response. Hypoxia and inflammation activation accompanied by the activation of the glycolysis pathway can increase the production and release of lactic acid. As a key metabolite in the glycolysis pathway, lactic acid can bind to MAVS (mitochondrial antiviral-signalling protein). The purpose is to prevent its localization to the mitochondria, inhibit the interaction with RIG-1, downstream signal transduction, and activation of type I interferon. In this way, the immune response is suppressed and the body’s ability to resist viruses is reduced (Zhang et al., 2019).

Our results indicate candidate genes in the liver were significantly enriched in the RIG-I-like receptor signaling pathway. Therefore, we suggest that in our experimental fish, the death of the Intol-group under acute hypoxia stress may be due to the accumulation of lactic acid caused by the activation of the early anaerobic glycolysis pathway, which reduces the immunity of the fish. In future work, a major research direction will be to find the activation mechanisms of various signaling pathways under hypoxia conditions in fish. The results presented here show that the brain and liver have a certain synergistic effect during hypoxia which promotes hypoxia adaptation.

## Conclusion

In this study, we used BSR-Seq analysis to explore the reasons for the differential hypoxia tolerance of the golden pompano population under acute hypoxia conditions. the arachidonic acid metabolism pathway in the brain of the Tol group may promote lipid metabolism and energy supply by regulating lipid decomposition, thereby improving the ability to adapt to hypoxia. However, the death of the Intol group under acute hypoxia stress may be due to the activation of the early anaerobic glycolysis pathway resulting in the accumulation of lactic acid, and the decrease of type I interferon production *in vivo*, which reduces the immunity of the fish. Therefore, the balance of glucose and lipid metabolism plays a key role in the hypoxic tolerance of fish under acute hypoxia stress*.* Although additional research is needed to confirm these possibilities, our findings suggest these SNPs will help carry out the molecular breeding of hypoxia-tolerant golden pompano, and at the same time confirm that under acute hypoxia stress, the balance of glucose and lipid metabolism plays a key role in the hypoxia tolerance of fish.

## Data Availability

The datasets presented in this study can be found in online repositories. The names of the repository/repositories and accession number(s) can be found below: https://www.ncbi.nlm.nih.gov/, GSE163685.
