# Supplementary figures and images for: Identification of Candidate Genes Associated With Hypoxia Tolerance in Trachinotus blochii Using Bulked Segregant Analysis and RNA-Seq
Source: Front Genet. 2021 Dec 14;12:811685. doi: 10.3389/fgene.2021.811685 (PMC8712738; doi:10.3389/fgene.2021.811685)

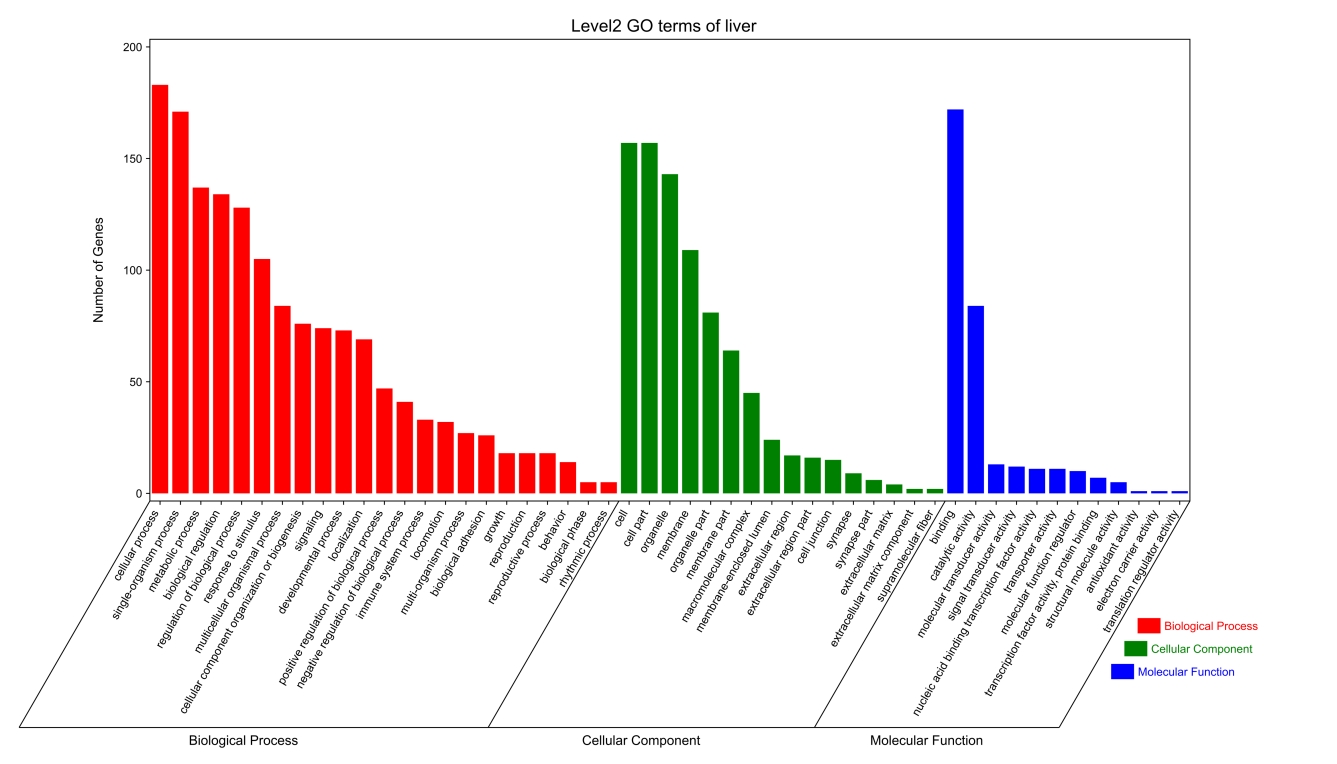

Supplement: Supplementary file 1 [file Image3.JPEG]

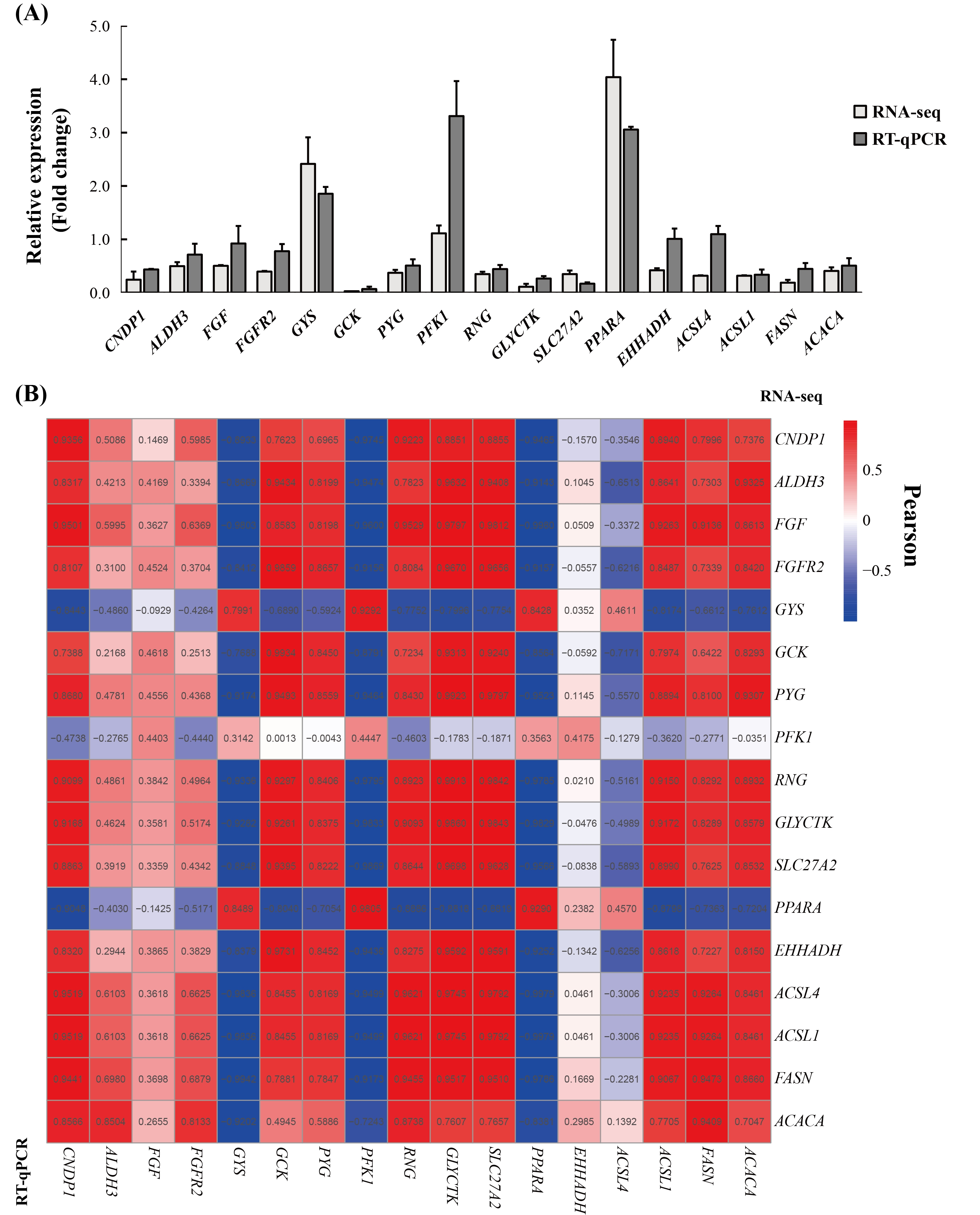

Supplement: Supplementary file 2 [file Image1.JPEG]

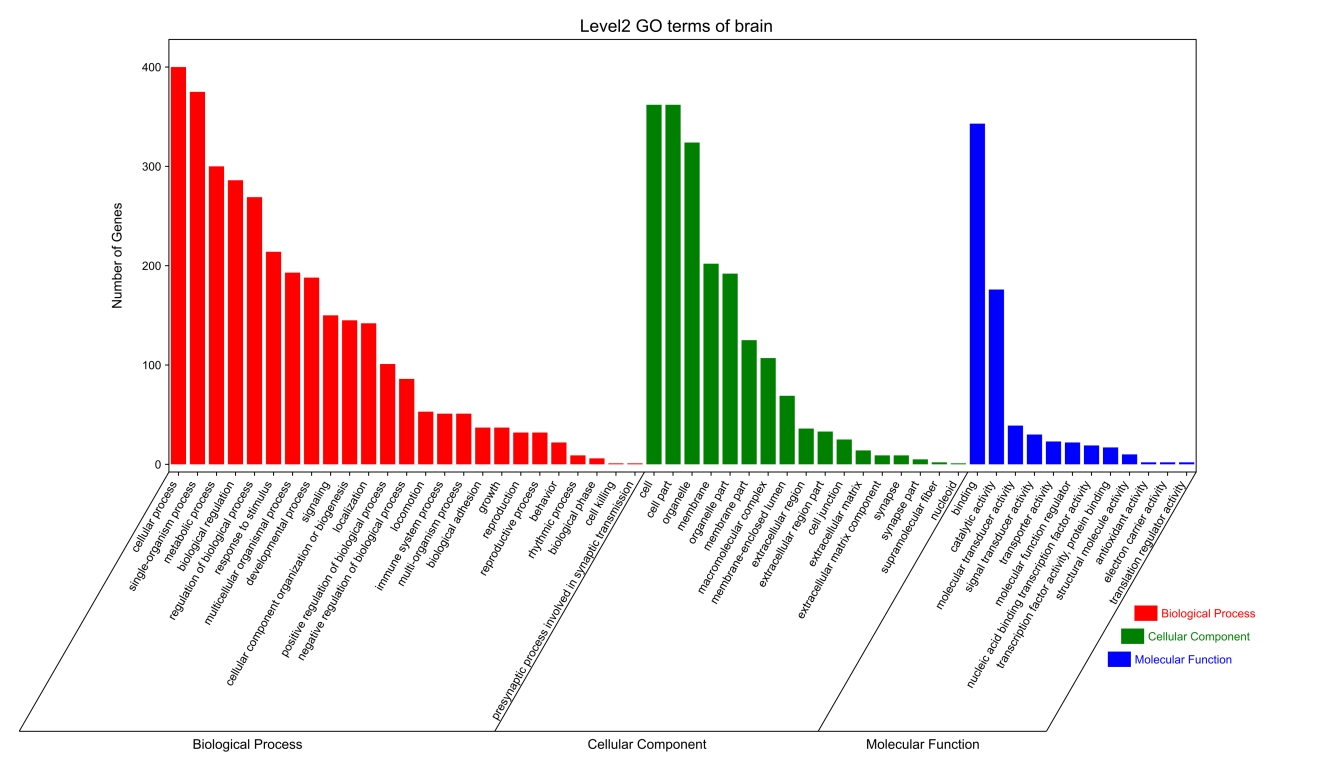

Supplement: Supplementary file 3 [file Image2.JPEG]
