# Supplementary material for: Identification of Candidate Genes Associated With Hypoxia Tolerance in Trachinotus blochii Using Bulked Segregant Analysis and RNA-Seq
Source: Front Genet. 2021 Dec 14;12:811685. doi: 10.3389/fgene.2021.811685 (PMC8712738; doi:10.3389/fgene.2021.811685)
Supplement: Supplementary file 4 [file Table1.DOC]

**Table S1 Primer information for RT-qPCR**

| **Gene** | **Primer (5'-3')** | | **TM (°C)** | **Product length (bp)** | **ID** |
| --- | --- | --- | --- | --- | --- |
| *CNDP1* | Forward primer | GCACAAAGACTGTCATCCCT | 58.4 | 228 | Trachinotus_GLEAN_10009079 |
|  | Reverse primer | TCTTGACAGCAGCCTTCCC | 58.5 |  |  |
| *ALDH3* | Forward primer | GACCCCATCATGCAACAGG | 58.5 | 235 | Trachinotus_GLEAN_10019575 |
|  | Reverse primer | CTACTCCACCGAAAGGCAGA | 57.6 |  |  |
| *FGF* | Forward primer | AAGACGCATTCACAGAGACT | 59.0 | 178 | Trachinotus_GLEAN_10018663 |
|  | Reverse primer | ATCTAACCAAATGCGCCAGA | 58.6 |  |  |
| *FGFR2* | Forward primer | TGCCAACACAGTCAAGTTCCG | 59.9 | 119 | Trachinotus_GLEAN_10017043 |
|  | Reverse primer | AGCGCACCTTATATCCTCCC | 59.5 |  |  |
| *GYS* | Forward primer | AGTTCTCAGCCATGCACGA | 59.4 | 144 | Trachinotus_GLEAN_10012093 |
|  | Reverse primer | ACTCATAACGTCCAGCGAT | 58.4 |  |  |
| *PYG* | Forward primer | ACGACGTTGATGCACTTGACA | 60.5 | 274 | Trachinotus_GLEAN_10001891 |
|  | Reverse primer | CCGGCAATGTTGTAGATCACC | 60.3 |  |  |
| *HKDC1* | Forward primer | GCTGCCATCTTGACTCGAA | 57.8 | 136 | Trachinotus_GLEAN_10015371 |
|  | Reverse primer | GAAGCAACCGACGCACCA | 58.0 |  |  |
| *GCK* | Forward primer | CTTCTCCTTTCCGGTACGACA | 59.1 | 144 | Trachinotus_GLEAN_10009126 |
|  | Reverse primer | ATCTCAAAGTCCCCTCGTCT | 59.3 |  |  |
| *PFK1* | Forward primer | AGAAAGGCATCACCAACCTG | 59.9 | 265 | Trachinotus_GLEAN_10006442 |
|  | Reverse primer | ACCAAGGCCAGATACCCACA | 60.5 |  |  |
| *RGN* | Forward primer | CTCTGACCTCTCCGTGACCA | 60.4 | 212 | Trachinotus_GLEAN_10002138 |
|  | Reverse primer | CTCCATTGTAACAGGCGACCC | 60.3 |  |  |
| *GLYCTK* | Forward primer | AACTTGCCTGACTCTGATGC | 58.5 | 172 | Trachinotus_GLEAN_10015968 |
|  | Reverse primer | GTCTGCGAGTAACATCCAGT | 57.8 |  |  |
| *SLC27A2* | Forward primer | TCAACCTGCCTCTGTACCAC | 58.0 | 266 | Trachinotus_GLEAN_10007926 |
|  | Reverse primer | TGAATGTTCCCAAAGCGACT | 57.3 |  |  |
| *PPARA* | Forward primer | ACGACAAGTGTGAACGCAAC | 59.8 | 290 | Trachinotus_GLEAN_10000221 |
|  | Reverse primer | CTTTCCAGTGAGTATGAGCCT | 59.2 |  |  |
| *EHHADH* | Forward primer | CTGCCTTAGACCTCATCACC | 57.3 | 112 | Trachinotus_GLEAN_10006450 |
|  | Reverse primer | AACTTTACAGCTGCATCCAC | 57.3 |  |  |
| *ACSL1* | Forward primer | CCATACACATCCACACCGAGT | 60.6 | 114 | Trachinotus_GLEAN_10004723 |
|  | Reverse primer | ACAGCTCTTCTATCTGGGGTC | 59.4 |  |  |
| *ACSL4* | Forward primer | AAGGACAAGCCGAACCCAA | 60.0 | 383 | Trachinotus_GLEAN_10005451 |
|  | Reverse primer | TCCTCCCATGCTCCAACGA | 59.9 |  |  |
| *FASN* | Forward primer | CAACCGGATACGCTGTGCAT | 61.8 | 144 | Trachinotus_GLEAN_10008918 |
|  | Reverse primer | AAGACACCCCAACGTCCATC | 61.6 |  |  |
| *ACACA* | Forward primer | ACGCCTCAGAGAACCCCAA | 61.5 | 383 | Trachinotus_GLEAN_10017921 |
|  | Reverse primer | AACTTCTGCCTGGACCTGT | 61.4 |  |  |
| *β*-actin | Forward primer | TACGAGCTGCCTGACGGACA | 63.0 | 240 | GenBank: MK250485.1 |
|  | Reverse primer | GGCTGTGATCTCCTTCTGCA | 60.0 |  |  |
