# Supplementary material for: Identification of Candidate Genes Associated With Hypoxia Tolerance in Trachinotus blochii Using Bulked Segregant Analysis and RNA-Seq
Source: Front Genet. 2021 Dec 14;12:811685. doi: 10.3389/fgene.2021.811685 (PMC8712738; doi:10.3389/fgene.2021.811685)
Supplement: Supplementary file 5 [file Table2.DOC]

**Table S2 Statistical results of the intersection of candidate genes in brain and liver**

| Gene ID |
| --- |
| Trachinotus_GLEAN_10003095; Trachinotus_GLEAN_10003096; Trachinotus_GLEAN_10008822;  Trachinotus_GLEAN_10008823; Trachinotus_GLEAN_10008824; Trachinotus_GLEAN_10008825;  Trachinotus_GLEAN_10008826; Trachinotus_GLEAN_10008827; Trachinotus_GLEAN_10008828;  Trachinotus_GLEAN_10008829; Trachinotus_GLEAN_10008830; Trachinotus_GLEAN_10008831;  Trachinotus_GLEAN_10008832; Trachinotus_GLEAN_10008833; Trachinotus_GLEAN_10008834;  Trachinotus_GLEAN_10008835; Trachinotus_GLEAN_10008836; Trachinotus_GLEAN_10008837;  Trachinotus_GLEAN_10014107; Trachinotus_GLEAN_10014108; Trachinotus_GLEAN_10014109;  Trachinotus_GLEAN_10014110; Trachinotus_GLEAN_10014111; Trachinotus_GLEAN_10014112;  Trachinotus_GLEAN_10014113; Trachinotus_GLEAN_10014114; Trachinotus_GLEAN_10014115;  Trachinotus_GLEAN_10014116; Trachinotus_GLEAN_10014117; Trachinotus_GLEAN_10014118;  Trachinotus_GLEAN_10014119; Trachinotus_GLEAN_10014120; Trachinotus_GLEAN_10014121;  Trachinotus_GLEAN_10014122; Trachinotus_GLEAN_10014123; Trachinotus_GLEAN_10014124;  Trachinotus_GLEAN_10014126; Trachinotus_GLEAN_10014127; Trachinotus_GLEAN_10014128;  Trachinotus_GLEAN_10014129; Trachinotus_GLEAN_10014130; Trachinotus_GLEAN_10014131;  Trachinotus_GLEAN_10014132; Trachinotus_GLEAN_10014133; Trachinotus_GLEAN_10014134;  Trachinotus_GLEAN_10014135; Trachinotus_GLEAN_10014136; Trachinotus_GLEAN_10014137;  Trachinotus_GLEAN_10014138; Trachinotus_GLEAN_10014139; Trachinotus_GLEAN_10014141;  Trachinotus_GLEAN_10014142; Trachinotus_GLEAN_10014143; Trachinotus_GLEAN_10014144;  Trachinotus_GLEAN_10014145; Trachinotus_GLEAN_10014146; Trachinotus_GLEAN_10014147;  Trachinotus_GLEAN_10014179; Trachinotus_GLEAN_10014180; Trachinotus_GLEAN_10014181;  Trachinotus_GLEAN_10014182; Trachinotus_GLEAN_10014183; Trachinotus_GLEAN_10014184;  Trachinotus_GLEAN_10014185; Trachinotus_GLEAN_10014186; Trachinotus_GLEAN_10014187;  Trachinotus_GLEAN_10014188; Trachinotus_GLEAN_10014189; Trachinotus_GLEAN_10014190;  Trachinotus_GLEAN_10014191; Trachinotus_GLEAN_10014192; Trachinotus_GLEAN_10014193;  Trachinotus_GLEAN_10014194; Trachinotus_GLEAN_10014196; Trachinotus_GLEAN_10014197;  Trachinotus_GLEAN_10014198; Trachinotus_GLEAN_10014199; Trachinotus_GLEAN_10014200;  Trachinotus_GLEAN_10014203; Trachinotus_GLEAN_10014204; Trachinotus_GLEAN_10014205;  Trachinotus_GLEAN_10014206; Trachinotus_GLEAN_10014207; Trachinotus_GLEAN_10014208;  Trachinotus_GLEAN_10014209; Trachinotus_GLEAN_10014210; Trachinotus_GLEAN_10014211;  Trachinotus_GLEAN_10014212; Trachinotus_GLEAN_10014213; Trachinotus_GLEAN_10014214;  Trachinotus_GLEAN_10014215; Trachinotus_GLEAN_10014216; Trachinotus_GLEAN_10014217; |

Attach Table S2

| Gene ID |
| --- |
| Trachinotus_GLEAN_10014218; Trachinotus_GLEAN_10014219; Trachinotus_GLEAN_10014220;  Trachinotus_GLEAN_10017002; Trachinotus_GLEAN_10017006; Trachinotus_GLEAN_10017007;  Trachinotus_GLEAN_10017008; Trachinotus_GLEAN_10017009; Trachinotus_GLEAN_10017010;  Trachinotus_GLEAN_10017012; Trachinotus_GLEAN_10017013; Trachinotus_GLEAN_10017014;  Trachinotus_GLEAN_10017015; Trachinotus_GLEAN_10017016; Trachinotus_GLEAN_10017017;  Trachinotus_GLEAN_10017018; Trachinotus_GLEAN_10017021; Trachinotus_GLEAN_10017022;  Trachinotus_GLEAN_10017023; Trachinotus_GLEAN_10017024; Trachinotus_GLEAN_10017025;  Trachinotus_GLEAN_10017026; Trachinotus_GLEAN_10017027; Trachinotus_GLEAN_10017028;  Trachinotus_GLEAN_10017029; Trachinotus_GLEAN_10017030; Trachinotus_GLEAN_10017031;  Trachinotus_GLEAN_10017032; Trachinotus_GLEAN_10017034; Trachinotus_GLEAN_10017035;  Trachinotus_GLEAN_10017037; Trachinotus_GLEAN_10017038; Trachinotus_GLEAN_10017042;  Trachinotus_GLEAN_10017043; Trachinotus_GLEAN_10017044; Trachinotus_GLEAN_10017045;  Trachinotus_GLEAN_10017046; Trachinotus_GLEAN_10017047; Trachinotus_GLEAN_10017048;  Trachinotus_GLEAN_10017049; Trachinotus_GLEAN_10017050; Trachinotus_GLEAN_10017051;  Trachinotus_GLEAN_10017052; Trachinotus_GLEAN_10017053; Trachinotus_GLEAN_10017054;  Trachinotus_GLEAN_10017055; Trachinotus_GLEAN_10017056 |
